# DiScRIBinATE: a rapid method for accurate taxonomic classification of metagenomic sequences

**DOI:** 10.1186/1471-2105-11-S7-S14

**Published:** 2010-10-15

**Authors:** Tarini Shankar Ghosh, Monzoorul Haque M, Sharmila S Mande

**Affiliations:** 1 Bio-Sciences Division, Innovation Labs, Tata Consultancy Services, 1 Software Units Layout, Hyderabad 500 081, Andhra Pradesh, India

## Abstract

**Background:**

In metagenomic sequence data, majority of sequences/reads originate from new or partially characterized genomes, the corresponding sequences of which are absent in existing reference databases. Since taxonomic assignment of reads is based on their similarity to sequences from known organisms, the presence of reads originating from new organisms poses a major challenge to taxonomic binning methods. The recently published SOrt-ITEMS algorithm uses an elaborate work-flow to assign reads originating from hitherto unknown genomes with significant accuracy and specificity. Nevertheless, a significant proportion of reads still get misclassified. Besides, the use of an alignment-based orthology step (for improving the specificity of assignments) increases the total binning time of SOrt-ITEMS.

**Results:**

In this paper, we introduce a rapid binning approach called DiScRIBinATE (Distance Score Ratio for Improved Binning And Taxonomic Estimation). DiScRIBinATE replaces the orthology approach of SOrt-ITEMS with a quicker 'alignment-free' approach. We demonstrate that incorporating this approach reduces binning time by half without any loss in the specificity and accuracy of assignments. Besides, a novel reclassification strategy incorporated in DiScRIBinATE results in reducing the overall misclassification rate to around 3 - 7%. This misclassification rate is 1.5 - 3 times lower as compared to that by SOrt-ITEMS, and 3 - 30 times lower as compared to that by MEGAN.

**Conclusions:**

A significant reduction in binning time, coupled with a superior assignment accuracy (as compared to existing binning methods), indicates the immense applicability of the proposed algorithm in rapidly mapping the taxonomic diversity of large metagenomic samples with high accuracy and specificity.

**Availability:**

The program is available on request from the authors.

## Background

The enormous microbial diversity prevalent in natural ecosystems represents a rich resource for discovery of hitherto unknown microbes and the novel genes/proteins they encompass. Estimates reveal that 99% of these microbes cannot be easily cultured in the laboratory [1]. The rapidly growing field of metagenomics directly investigates this microbial diversity by obtaining and sequencing the entire genomic content present in any given environmental sample. Since environmental samples contain hundreds of microbes, the sequencing step typically generates millions of sequenced fragments originating from the genomes of various microbes. Using computational methods, these sequences are subsequently analysed to identify new organisms, genes, proteins, and metabolic pathways.

An important aspect in metagenomic analysis is the identification of the source organism of each fragment (read/contig). This process, called binning, helps in identifying and enumerating the various taxa present in a given metagenomic sample. Binning approaches can be broadly classified into two categories, namely, composition-based and similarity-based. Composition-based approaches cluster and classify sequences based on their compositional characteristics, such as GC percent, codon usage, oligo-nucleotide frequency distribution, etc. [2,3]. On the other hand, similarity-based approaches identify sequence(s) in reference databases, which display significant similarity with the read sequence [4,5].

In similarity-based approaches, sequences in the database having significant similarity to the input reads are referred to as hits. While a read with hit(s) originating from a single organism is assigned to the organism corresponding to the hit(s), a read with hits originating from multiple organisms is assigned to the Lowest Common Ancestor (LCA) of the taxa which correspond to these organisms. The specificity of assignment using the LCA approach is thus dependent on the spatial distribution of hits in the phylogenetic tree.

To improve the specificity of assignment of reads, similarity-based methods like MEGAN use bit-score of an alignment to identify a subset of hits which are significant, and subsequently assign the read to the LCA of this subset [4]. However, the MEGAN approach was seen to have a significantly high false positive rate and low specificity of assignments, especially in scenarios where reads originated from new organisms [5]. These limitations have been addressed by SOrt-ITEMS algorithm, which adopts a two-phase binning approach [5]. During the first phase, additional alignment parameters (identities, positives and the bit-score) are used by SOrt-ITEMS to ascertain the quality of the hits obtained for a read. Reads having hits with high quality alignments are allowed to be assigned at specific taxonomic levels such as species, genus or family. In contrast, the assignment of reads which generate alignments of lower quality is restricted to relatively higher taxonomic levels. Thus, the better the quality of the alignment, the more specific is the taxonomic level of assignment, and *vice-versa*. Once this level is identified, SOrt-ITEMS employs the 'orthology step' in the second phase to finally assign the read to the LCA of the taxa which correspond to the subset of hits that share a true orthologous relationship with the read sequence. While it is seen that the first phase of SOrt-ITEMS helps in significantly reducing the number of false positive assignments, the second phase ensures the specificity of assignments *i.e*. assignment of reads at relatively lower taxonomic levels (family, genus or species).

In spite of having significantly higher binning specificity and accuracy, the SOrt-ITEMS approach has the following two limitations. Firstly, since SOrt-ITEMS involves performing a reciprocal BLAST search, an additional time is spent (for every read) in this 'orthology step'. Summing this additional time for all reads in a typical metagenomic data set (having approximately 1-10 million sequences) results in a significant increase in the total computation time. Secondly, about 7-10% of input sequences still get misclassified by SOrt-ITEMS. This misclassification rate is significant since this will result in several thousands of sequences being classified wrongly. For example, if one analyzes the Sargasso sea metagenome data set consisting of greater than 7 million sequences [6], approximately 300,000 to 700,000 sequences are likely to be assigned to incorrect taxa. These wrong assignments will definitely impact the accuracy of several downstream analyses.

One way of reducing the number of misclassified sequences is by increasing the threshold of the 'minimum bin-size' parameter (*i.e *the minimum number of sequences to be assigned to a taxon for a bin to be created for that respective taxon). In this process, assignments to isolated taxa (*i.e*. taxa to which the number of sequences assigned is less than the minimum bin-size threshold) are discarded. Increasing the value of this threshold parameter, will thus reduce the number of false positive assignments. However this strategy will result in an increased number of unassigned sequences.

In this paper, we propose a new approach termed as DiScRIBinATE (Distance Score Ratio for Improved Binning And Taxonomic Estimation) which attempts to address the limitations associated with SOrt-ITEMS. To maintain the accuracy of assignments, DiScRIBinATE retains the steps followed in the first phase of SOrt-ITEMS i.e. for finding an appropriate taxonomic level where the assignment of the read is to be restricted.

However, to ensure the specificity of assignments, DiScRIBinATE circumvents the time consuming 'orthology' step of SOrt-ITEMS by utilizing a quicker 'alternative approach' based on the ratio of bit-score and distance information obtained from the hits corresponding to a read. We demonstrate that the proposed 'alternative approach' significantly decreases the time taken for binning by half. Besides, a novel reclassification strategy incorporated in DiScRIBinATE reduces the misclassification rate to around 3 - 7%. This misclassification rate is around 1.5 - 3 times lower than that by SOrt-ITEMS and 3 - 30 times lower as compared to MEGAN. We also demonstrate that adopting this novel strategy does not increase the percentage of unassigned reads.

## Results

### DiScRIBinATE algorithm

The DiScRIBinATE approach takes the BLASTx output obtained for all the reads against a sequence database (*e*.*g *nr database) as the input [7]. The appropriate taxonomic level (TL) where the assignment of each read needs to be restricted is identified using the approach followed by SOrt-ITEMS [5]. This is done by analysing various alignment parameters obtained using the best hit for a given read. The DiScRIBinATE approach uses the same thresholds of these alignment parameters as used in SOrt-ITEMS [5]. Once TL is identified, the taxon name of the best hit is replaced by the taxon name at TL. The taxon names of other hits are also substituted with the respective taxa names that occur at TL. For example, if the TL of a read lies at the level of family, the taxon name obtained for the best hit (*e.g Burkholderia ambifaria *AMMD) is substituted by the name of the corresponding family (*i.e*, Burkholderiaceae). Similarly, taxon names of the remaining hits are also substituted with their corresponding taxa names that occur at the family level. These substituted names are used in all subsequent steps. Therefore, the taxonomic level of the final assignment of the read can only occur either at the TL or at levels above TL, *i.e*. to a taxon in the phylogenetic path from the TL to the root. For tracing the path between the root to the taxon name corresponding to each hit, the NCBI taxonomy tree has been utilized as the reference tree.

**Figure 1:**
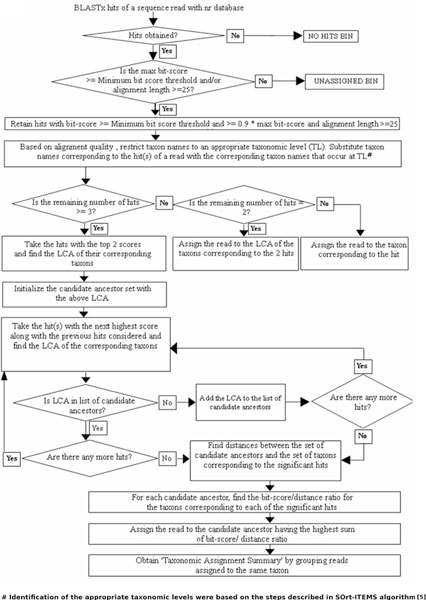
**Steps of the DiScRIBinATE algorithm**. Flowchart illustrating the steps followed by DiScRIBinATE for the Bit-score/Distance ratio based assignment of reads (Steps 1-3).

**Figure 2:**
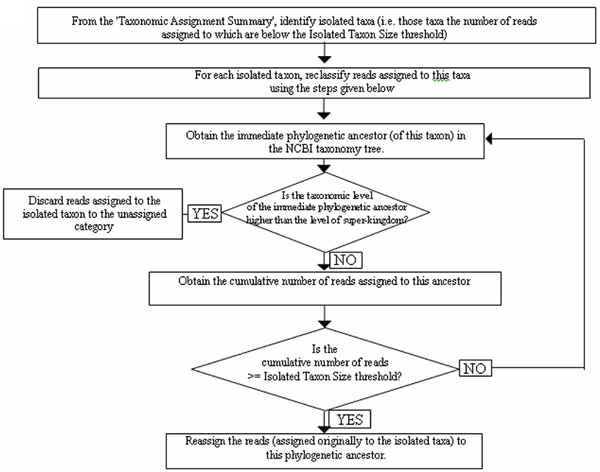
**The reclassification steps of the DiScRIBinATE algorithm**. Flowchart illustrating the reclassification steps followed by DiScRIBinATE (Step 4).

Once the taxa names corresponding to the hits are substituted with the corresponding taxa names occurring at their respective TLs, reads with only one hit are assigned to the substituted taxon/clade corresponding to the hit. If a read has two hits, it is assigned to the LCA of the taxa/clades corresponding to these two hits. However, for reads with three or more hits, the following steps are performed for the final assignment of the read. The flowchart illustrating the first three steps of the 'DiScRIBinATE' work-flow is given in Figure 1 and that of the last step (step 4) is given in Figure 2.

#### Step 1. Obtaining a set of candidate ancestors

The nodes of the NCBI taxonomic tree follow a typical parent-child hierarchy, wherein the descendant nodes (intermediate or terminal) are referred to as 'child nodes'. All child nodes unidirectionally descend from the 'root node'. Intermediate nodes, which lie in the path from the root to the child, are referred to as 'ancestor nodes' for the corresponding child node. The set of unique candidate ancestors corresponding to each read is progressively obtained by finding the LCA of each of the substituted taxa names as explained below.

Among all the hits obtained for a given read, first the LCA of the taxon names corresponding to the top two scoring hits is obtained. This LCA is identified as the first candidate ancestor. The hit with the next highest score is then added to the previous hits considered, and the combined LCA of the corresponding taxa names is obtained from these hits. If this LCA is not the same as the one obtained earlier, it is added to the existing set of candidate ancestors. In this way a set of candidate ancestors for a given read is obtained by traversing the entire list of hits till the last hit is reached.

#### Step 2. Obtaining distances between candidate ancestors and hits

In this step, distances are calculated between the candidate ancestors and the set of taxa corresponding to the hits. The level of a taxon in the NCBI taxonomy tree denotes the distance traversed in terms of the number of edges from the root to that taxon. This level information is used for obtaining distances between any two taxa. If the taxon corresponding to a hit is a child node of a candidate ancestor, then the distance between the candidate ancestor and that taxon is equal to difference of levels between them. If the taxon corresponding to a hit is not a child node of a candidate ancestor, then the LCA of the candidate ancestor and that taxon is obtained, and the distance between them is subsequently calculated using the following formula:

$$\begin{array}{c} \text{Distance}=\text{Distance}\;\text{between the LCA and Candidate ancestor}+\text{Distance between the}\\ \text{LCA and the taxon corresponding to the hit}\\ =\text{ [Level (Candidate ancestor)}-\text{Level(LCA)]}+[\text{Level(taxon corresponding to the hit)}-\text{Level(LCA)}] \end{array}$$

#### Step 3. Assignment of reads to the 'Most Probable Ancestor'

In this step, the 'bit-score/distance' ratio between a hit and a candidate ancestor is obtained by dividing the bit-score of the hit by the distance of its corresponding taxon from the candidate ancestor (obtained as described in the previous step). These ratios are obtained considering all the hits and the candidate ancestors. For each candidate ancestor, the 'bit-score/distance' ratios obtained with each of the hits are then summed. The candidate ancestor with the highest sum, referred to as the 'Most Probable Ancestor' (MPA), is selected, and the read is assigned to the taxon corresponding to this candidate ancestor.

#### Step 4. Re-classifying assignments to isolated taxa

The objective of this step is to reduce the number of misclassified sequences. For this purpose, all assignments to isolated taxa are re-analyzed using the work-flow illustrated in Figure 2. Isolated taxa are defined as those taxa to which the number of reads assigned is less than a given threshold. This threshold is hereafter referred to as 'Isolated Taxon Size'.

In this step, reads assigned to each isolated taxon are reassigned to the taxon corresponding to their immediate phylogenetic ancestor, if the cumulative number of reads assigned to the latter taxon is greater than the Isolated Taxon Size. If this cumulative number does not exceed the Isolated Taxon Size, the above mentioned step is iterated for progressively higher taxonomic levels (up to the level of super-kingdom). This iteration is performed till an ancestor taxon is found for which the cumulative number of reads assigned exceeds the Isolated Taxon Size. However, if the cumulative number of reads for any of the ancestor taxa does not exceed the 'Isolated Taxon Size', reads assigned to the isolated taxon are categorized as 'unassigned'.

In the present study, an 'Isolated Taxon Size' of 300 or 1% of the total number of reads (whichever is less) was used. A value of 300 was used keeping in mind the presence of rare organisms in typical metagenomic samples. This value ensured that reads originating from rare organisms or organisms with small genome sizes are not unnecessarily reassigned at non-specific taxonomic levels. For example, the value of 300 ensures that an organism having a genome length as low as 0.6 Mb and also with extremely low coverage (as low as 0.5X, 0.2X, 0.13X and 0.05X, for read lengths of 1000 bp, 400 bp, 250 bp and 100 bp, respectively) is not picked up as an isolated taxon.

Consequently, reads belonging to this organism are not re-assigned at non-specific taxonomic levels during the reclassification step.

### Strategy for evaluating the binning time

In order to evaluate the binning time achieved using the 'bit-score/distance ratio approach' (adopted by DiScRIBinATE) as compared to the 'orthology based approach' (used by SOrt-ITEMS) and standard LCA-based approach (used by MEGAN), the time taken by DiScRIBinATE, SOrt-ITEMS and MEGAN for binning 100000, 200000, 500000 and 1000000 sequences was determined. These tests were performed on a work station with Intel(R) Xeon(R) CPU, 1.86 GHz processor.

### Data sets used for evaluating binning accuracy and specificity

Thirty-five completely sequenced bacterial genomes belonging to diverse taxonomic clades were downloaded from NCBI website ftp://ftp.ncbi.nih.gov/genomes/Bacteria/. Using MetaSim [8], four data sets were created namely, Sanger, 454-400, 454-250 and 454-100. Each data set consisted of 35,000 reads (1000 reads from each genome) simulating the typical read lengths and errors models associated with Sanger (read lengths centred round 800 base pairs), 454-Titanium (400 bp), 454-FLX (250 bp), and 454-GS20 (100 bp) respectively.

### Database variants used for evaluating binning accuracy and specificity

To validate the performance of DiScRIBinATE, with respect to the assignment of reads originating from 'known' as well as 'unknown' organisms, input reads were queried against the following three variants of the nr database.

#### a. 'Species unknown'

nr database where sequences belonging to the query species are absent. This scenario mimics a scenario wherein the read sequences belong to an unknown species.

#### b. 'Genus unknown'

nr database where sequences belonging to the query genus are absent. This scenario simulates a situation wherein read sequences originate from an unknown genus.

#### c. 'Family unknown'

nr database where sequences belonging to the query family are absent. This scenario simulates a situation wherein read sequences originate from an unknown family.

### Categorization of taxonomic assignments

To quantify the accuracy and specificity of assignment of reads, the results obtained for the four validation data sets against the three database variants (mentioned above) were categorized as follows:

#### a. Correct assignments

Assignment of a read to a taxon that lies in the path between the root and the taxon corresponding to the source organism of the read was categorized as 'correct assignment'. For example, if the read originated from *Burkholderia ambifaria *AMMD, then its assignment to any of the taxa mentioned below was categorized as a correct assignment.

root; cellular organisms; Bacteria; Proteobacteria; Betaproteobacteria; Burkholderiales; Burkholderiaceae; *Burkholderia; Burkholderia cepacia *complex; *Burkholderia ambifaria; Burkholderia ambifaria *AMMD.

To quantify the specificity, the 'Correct assignments' were sub-grouped into the following categories:

*1. Higher (or Non-Specific) level*: Correct assignments of the reads at the level of root or cellular organisms or super-kingdom.

*2. Intermediate levels*: Correct assignments of the reads at the level of phylum or class or order.

*3. Specific levels*: Assignments of the reads at the level of family or below.

#### b. Wrong assignments

Assignment of a read to a taxon that does not lie in the path between the root and the taxon corresponding to the source organism of the read was categorized as 'Wrong assignment'.

#### c. Unassigned

Those reads for which none of the hits had a bit-score greater than or equal to 35 and/or an alignment length of greater than 25 were classified as 'Unassigned'.

#### d. No hits

All reads with no BLAST hits were categorized as 'No hits'.

Results obtained using DiScRIBinATE were categorized into the above classes and were compared with corresponding results generated by the SOrt-ITEMS and the MEGAN program. Both SOrt-ITEMS and MEGAN were run using a "minimum bin-size" threshold of two. Results obtained for DiScRIBinATE after applying the reclassification step were compared with those obtained with SOrt-ITEMS and MEGAN.

### Validation results

#### Comparative evaluation of binning time

**Figure 3:**
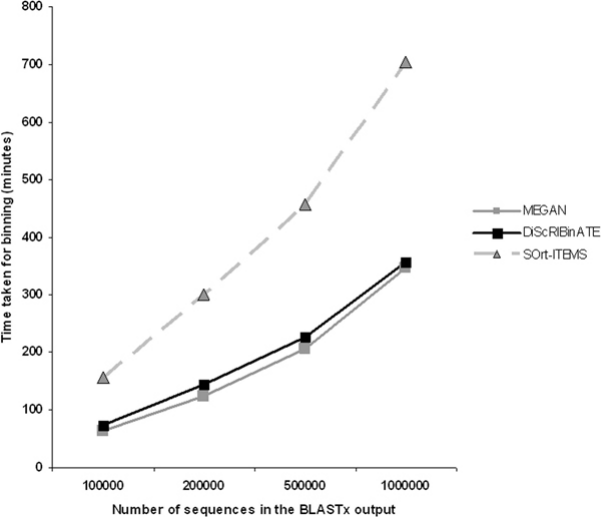
**Comparative evaluation of binning time**. Plot comparing the time taken (in minutes) by DiScrIBinATE (black-solid line), MEGAN (grey-solid line) and SOrt-ITEMS (grey-dotted line) for binning 100000, 200000, 500000 and 1000000 metagenomic reads. The tests were performed on an Intel (R) Xeon CPU workstation (with a 1.86 Ghz processor).

As shown in Figure 3, DiScrIBinATE takes just half the time (similar to MEGAN), as compared to SOrt-ITEMS, for binning an equivalent number of reads. This is expected since the 'bit-score/distance ratio' approach adopted in DiScRIBinATE is an alignment-free method and involves simple mathematical calculations, as against the orthology step (involving alignment of sequences) used in SOrt-ITEMS.

#### Comparative evaluation of binning accuracy and specificity

**Figure 4:**
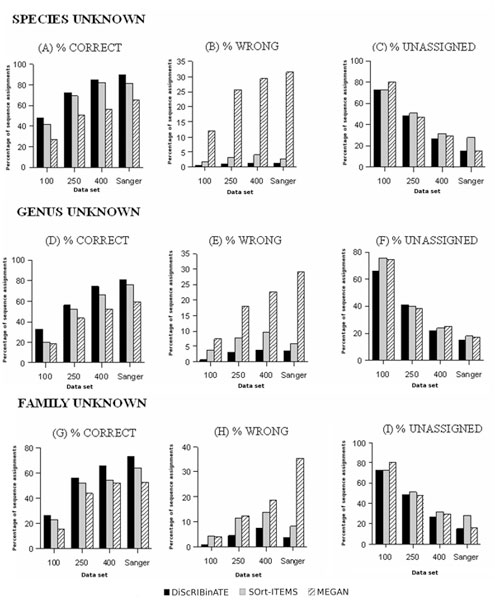
**Comparative evaluation of binning accuracy**. Graphical representation of results (for all data sets/database scenarios) showing the percentage of sequence assignments categorized as Correct, Wrong and Unassigned respectively (obtained for the three methods).

Figure 4 and Additional file 1 summarize results obtained by DiScRIBinATE, MEGAN and SOrt-ITEMS for all four validation data sets using three database variants. For the current validation study, the three programs were run with the Minimum Bit-Score threshold of 35. SOrt-ITEMS and MEGAN were run with a minimum bin size (min-support) value of two.

As seen from Figure 4, the percentage of 'correct' assignments is consistently higher for DiScRIBinATE as compared to SOrt-ITEMS and MEGAN (Figure 4A, D, G). Moreover, this pattern is observed to be consistent across all the data sets and database variants. While, DiScRIBinATE has the lowest misclassification rate across all data sets and database variants, MEGAN has the highest percentage of reads being misclassified (Figure 4B, E, H). On the other hand, percentage of reads being categorized as 'Unassigned' is roughly similar for all three programs across all data sets/database scenarios (Figure 4C, F, I). The detailed results of assignments to various bin categories are summarized below:

##### Correct assignments

The percentage of reads correctly assigned by DiScRIBinATE is seen to be 2-18% higher as compared to SOrt-ITEMS, and 11 - 30% higher as compared to MEGAN. Interestingly, this quantum of difference becomes higher and noticeable as sequences belonging to progressively higher clade levels are removed from the reference database (e.g. results with 'Genus/Family unknown' database variants). Given the composition of typical metagenomic data sets (where majority of sequences originate from hitherto unknown taxa/clades), results obtained with DiScRIBinATE (especially with Genus/Family unknown database variants) indicate its suitability for use with metagenomic sequence data sets.

##### Specific levels

Results (Additional file 1) indicate that the percentage of reads assigned by DiScRIBinATE and SOrt-ITEMS at specific levels is higher than that of MEGAN for the 454 data sets/database variants. However, MEGAN assigns a higher percentage of reads in the Sanger data set at specific levels as compared to DiScRIBinATE and SOrt-ITEMS. The likely reason for the latter observation is the following. Since, the Sanger data set has reads with a higher length range (600-1100 bp), the hits generated for these reads generally have higher bit-scores. Besides, it is observed that the best hits (obtained for a read) generally have a bit-score which is relatively much higher than the remaining hits (obtained for that read). Applying the top percent criteria (of MEGAN) therefore results in very few hits remaining for analysis. Consequently, the LCA tends to converge at relatively specific taxonomic levels. This resulting in higher specificity for some of the assignments.

However, it should be noted that higher bit-scores do not necessarily correspond to higher alignment quality. Consequently, MEGAN's approach is specific and accurate only in cases where the best hits are from a correct taxon/clade. However, in typical metagenomic scenarios, majority of query sequences originate from hitherto unknown organisms. In such scenarios, the best hits for a majority of query sequences are usually from an incorrect taxon/clade. Using MEGAN's LCA approach in such scenarios will naturally lead to incorrect assignments. This is evident from our results (see later sections) wherein MEGAN, in spite of having a higher specificity in the case of SANGER reads, has a mis-classification rate which is 3 - 30 times higher than DiScRIBinATE and SOrt-ITEMS.

It is also observed that the binning specificity of DiScRIBinATE is slightly lower as compared to SOrt-ITEMS. This marginal decrease of specificity (1-2%) can be attributed to the reclassification step used in DiScRIBinATE, wherein assignments to isolated taxa are moved to relatively higher taxonomic levels.

##### Intermediate levels

The percentage of reads assigned by DiScRIBinATE at intermediate levels is around 4-10% higher than SOrt-ITEMS, and around 9-40% higher than MEGAN (Additional File 1). This is expected since the reclassification step in DiScRIBinATE essentially reassigns reads (those which were earlier assigned to isolated taxa) at progressively higher taxonomic levels. Since assignments to such isolated taxa are usually found to be false positive assignments, the reclassification step facilitates their reclassification (albeit at slightly higher taxonomic levels), instead of discarding them as 'unassigned'.

##### Higher levels

Except for the 454-100 data set, it is seen that MEGAN assigns a higher percentage of reads at higher levels, as compared to DiScRIBinATE or SOrt-ITEMS. As compared to SOrt-ITEMS, DiScRIBinATE is seen to assign a marginally higher percentage of reads at higher levels. This marginal increase is a likely result of the reclassification step adopted by DiScRIBinATE.

##### Wrong assignments

As previously mentioned, the misclassification rate of DiScRIBinATE is significantly lower than both SOrt-ITEMS and MEGAN. Results indicate that MEGAN has the highest misclassification rate. Interestingly, as the lengths of the input reads become smaller, there is a progressive increase in the misclassification rate by SOrt-ITEMS and MEGAN as compared to that by DiScRIBinATE. Overall, the misclassification rate of DiScRIBinATE is seen to be around 3 - 7%, which is 1.5 - 3 times lower as compared to that by SOrt-ITEMS, and 3 - 30 times lower as compared to that by MEGAN. The above observations demonstrate the immense utility of the reclassification step in reducing the percentage of incorrect assignments.

##### Unassigned

The percentage of reads categorized as 'unassigned' by all the three methods is comparable across all the data sets/database variants (Figure 4, Additional File 1).

## Discussion

Evaluation of similarity-based binning approaches (using simulated metagenomic data sets) have indicated that a significant percentage of reads get misclassified or are categorized as unassigned [4,5]. This is expected since a majority of reads in metagenomic data sets originate from new or partially characterized genomes, the sequences of which are absent in existing reference databases. Consequently, a majority of metagenomic sequences generate poor alignments with sequences in reference databases. The SOrt-ITEMS algorithm could accurately assign reads having poor alignment quality [5]. The SOrt-ITEMS algorithm ensured accuracy by adopting an elaborate work-flow for judging alignment quality before taxonomic assignment. In spite of this elaborate work-flow, validation studies indicated that a sizeable fraction of reads (7-10%) still get misclassified. This is due to the fact that some reads generate stray, yet significant hits (*i.e *with good alignment quality), with incorrect (and generally isolated) taxa present in reference databases. It is due to this reason that even an algorithm like SOrt-ITEMS (which takes in account the alignment quality before taxonomic assignment) assigns such reads to incorrect taxa. The latter reads (assigned to incorrect taxa) are manifested in the output as isolated assignments. One way of reducing the number of misclassified sequences is by discarding such isolated assignments. However, adopting this strategy will results in increased number of sequences categorized as unassigned.

In the current study, a novel reclassification strategy has been devised that attempts to reclassify assignments to isolated taxa instead of simply discarding them. The premise of this strategy is the following. The SOrt-ITEMS algorithm [5] revealed that over-binning (incorrect assignments to taxa that are related to the source taxon only at a higher taxonomic level) was one of the primary causes of misclassification.

Hence, it is likely that most of the assignments to isolated taxa could also be a result of over-binning. Therefore to improve binning accuracy, the method presented in this paper attempts to re-classify assignments to isolated taxa at progressively higher taxonomic levels, rather than simply discarding them. The results obtained in this study have revealed that adopting this strategy significantly reduces the percentage of wrong assignments without increasing the percentage of unassigned reads.

The method described in this paper also incorporates a quicker 'alternative approach' (as compared to the time consuming orthology approach used by SOrt-ITEMS) based on the ratio of bit-score and distance information. The use of such a ratio (for improving specificity) is based on the following premise. Bit-score is one of the parameters in the BLAST output which reflects the overall quality of an alignment. Higher bit-scores indicate higher similarity between the query and the hit sequences. Furthermore, higher similarity also indicates that the organism corresponding to the query and hit sequences are taxonomically close to each other (i.e. the phylogenetic distance between them is low). Hence a ratio of bit-score/distance is directly indicative of the taxonomic relatedness.

**Figure 5:**
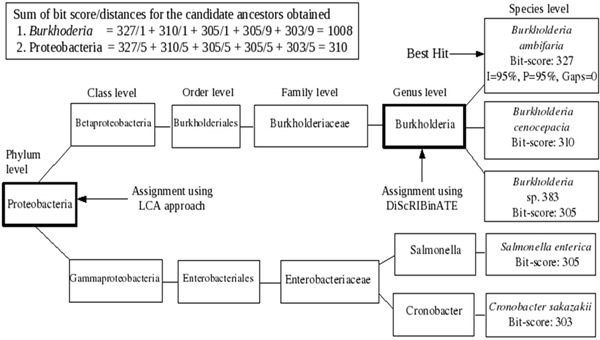
**Example illustrating the improved binning specificity obtained using 'bit-score/distance' approach**. An example illustrating the improved specificity of assignment using the 'bit-score/distance' ratios. In this example, the read originates from *Burkholderia ambifaria *(the corresponding sequences of which are present in the reference database). Based on the alignment parameters (Identities:I, Positives:P, Gaps) obtained for the best hit, the appropriate taxonomic level of assignment is restricted at the 'Species level'. Identified candidate ancestors are depicted in bold boxes. The read is assigned by DiScRIBinATE at the genus level (i.e. *Burkholderia*) on account of higher cumulative 'bit-score/distance' ratio. An LCA based approach would have assigned the same read at the phylum level (i.e Proteobacteria).

DiScRIBinATE uses the above principle to improve the specificity of assignments. For each query sequence, DiScRIBinATE identifies a set of candidate ancestors (from amongst the set of significant hits) and assigns the query to a candidate ancestor having the highest bit-score/distance ratio. Using this approach ensures that the query sequence is assigned to a taxon that is taxonomically closest to it (thereby improving specificity). The specificity of assignments using this alternate approach is seen to be comparable to those obtained using the orthology based approach. Figure 5 illustrates an example which demonstrates the process by which this alternative approach helps in improving the specificity of assignments. It is interesting to note that a similar approach had been used earlier for the identification of horizontal gene transfer in metagenomic samples [9].

Results indicate that, as compared to the orthology based approach, this alternative 'bit-score/distance approach', coupled with the reclassification step reduces the binning time by half and also significantly reduces the percentage of wrong assignments. As demonstrated in the validation results, the scale of reduction is as high as 30 times.

The three taxonomic binning methods described in this study use the output of a BLAST search as input. However, it is important to note that the time spent on the taxonomic assignment from BLAST results is really a tiny proportion as compared to the time spent on BLAST search. Besides developing efficient methods that can rapidly and accurately derive taxonomic inferences from BLAST outputs, it is also necessary to develop approaches that can reduce the time spent on performing the actual BLAST search. Our current research focus is on development of novel methods that reduce the computational time associated with typical BLAST searches.

## Conclusions

A significant reduction in binning time, coupled with a superior assignment accuracy (as compared to existing binning methods), indicates the immense applicability of the proposed algorithm in rapidly mapping the taxonomic diversity of large metagenomic samples with high accuracy and specificity.

## List of abbreviations used

SOrt-ITEMS: Sequence orthology approach for Identification and Taxonomic estimation of metagenomic sequences; LCA: Lowest Common Ancestor; MPA: Most Probable Ancestor; DiScRIBinATE: Distance Score Ratio for Improved Binning And Taxonomic Estimation; MEGAN: Metagenomic analyzer.

## Competing interests

The authors declare that they have no competing interests.

## Authors' contributions

TSG, MMH and SSM have conceived the idea and designed the detailed methodology. TSG and MMH have implemented the algorithm, created validation datasets and carried out detailed validation and testing of the algorithm. TSG, MMH and SSM have analyzed the data and finally drafted the complete paper.

## Supplementary Material

Additional file 1 **Table S1: Detailed summary of the percentage of assignments by DiScRIBinATE, SOrt-ITEMS and MEGAN under the various bin categories**. Click here for file
