# Supplementary material for: DiScRIBinATE: a rapid method for accurate taxonomic classification of metagenomic sequences
Source: BMC Bioinformatics. 2010 Oct 15;11(Suppl 7):S14. doi: 10.1186/1471-2105-11-S7-S14 (PMC2957682; doi:10.1186/1471-2105-11-S7-S14)
Supplement: Additional file 1 — Table S1: Detailed summary of the percentage of assignments by DiScRIBinATE, SOrt-ITEMS and MEGAN under the various bin categories. [file 1471-2105-11-S7-S14-S1.pdf]

**Additional file 1: Table S1:** Comparison of the percentage of reads assigned under various bin categories by DiScRIBinATE (DI), SOrt-ITEMS (SI) and MEGAN (ME) for the (A) 454-100 datasets (B) 454-250 datasets (C) 454-400 datasets and (D) Sanger datasets.

(A) 454-100

| ASSIGNMENT CATEGORIES | SPECIES UNKNOWN | | | GENUS UNKNOWN | | | FAMILY UNKNOWN | | |
|---|---|---|---|---|---|---|---|---|---|
| | DI | SI | ME | DI | SI | ME | DI | SI | ME |
| HIGHER LEVELS | 7.9 | 3.5 | 5.8 | 9.8 | 5.2 | 7.7 | 10.3 | 11.2 | 8.2 |
| INTERMEDIATE LEVELS | 20.3 | 15.4 | 7.3 | 19.4 | 10.1 | 8.3 | 16.0 | 11.7 | 7.2 |
| SPECIFIC LEVELS | 20.3 | 22.9 | 14.0 | 3.9 | 5.1 | 2.2 | 0.0 | 0.0 | 0.0 |
| TOTAL CORRECT ASSIGNMENTS | 48.5 | 41.8 | 27.1 | 33.1 | 20.4 | 18.2 | 26.3 | 22.8 | 15.4 |
| WRONG | 0.4 | 1.7 | 12.1 | 0.7 | 3.9 | 7.3 | 1.0 | 4.4 | 4.1 |
| UNASSIGNED + NO HITS | 51.1 | 56.6 | 60.8 | 66.2 | 75.7 | 74.4 | 72.7 | 72.8 | 80.6 |

(B) 454-250

| ASSIGNMENT CATEGORIES | SPECIES UNKNOWN | | | GENUS UNKNOWN | | | FAMILY UNKNOWN | | |
|---|---|---|---|---|---|---|---|---|---|
| | DI | SI | ME | DI | SI | ME | DI | SI | ME |
| HIGHER LEVELS | 4.9 | 6.0 | 10.8 | 11.6 | 10.5 | 19.0 | 13.3 | 11.5 | 18.8 |
| INTERMEDIATE LEVELS | 37.1 | 31.7 | 14.2 | 38.0 | 35.4 | 19.4 | 33.7 | 25.9 | 16.8 |
| SPECIFIC LEVELS | 30.4 | 31.9 | 25.9 | 6.4 | 6.2 | 5.3 | 0.0 | 0.0 | 0.0 |
| TOTAL CORRECT ASSIGNMENTS | 72.4 | 69.6 | 50.9 | 56.0 | 52.1 | 43.7 | 46.9 | 37.4 | 35.6 |
| WRONG | 1.1 | 3.1 | 25.6 | 3.1 | 7.8 | 17.8 | 4.7 | 11.7 | 12.6 |
| UNASSIGNED + NO HITS | 26.4 | 27.3 | 23.4 | 40.9 | 40.2 | 38.5 | 48.4 | 50.9 | 47.5 |

(C) 454-400

| ASSIGNMENT CATEGORIES | SPECIES UNKNOWN | | | GENUS UNKNOWN | | | FAMILY UNKNOWN | | |
|---|---|---|---|---|---|---|---|---|---|
| | DI | SI | ME | DI | SI | ME | DI | SI | ME |
| HIGHER LEVELS | 8.2 | 7.3 | 11.3 | 18.0 | 13.9 | 21.9 | 23.2 | 17.4 | 28.6 |
| INTERMEDIATE LEVELS | 42.3 | 38.8 | 14.2 | 48.4 | 44.4 | 23.5 | 42.4 | 36.9 | 22.8 |
| SPECIFIC LEVELS | 34.7 | 36.4 | 31.0 | 8.1 | 8.1 | 6.7 | 0.0 | 0.0 | 0.0 |
| TOTAL CORRECT ASSIGNMENTS | 85.2 | 82.5 | 56.5 | 74.5 | 66.4 | 52.1 | 65.6 | 54.3 | 51.4 |
| WRONG | 1.4 | 4.0 | 29.3 | 3.8 | 9.7 | 22.7 | 7.5 | 14.0 | 18.8 |
| UNASSIGNED + NO HITS | 13.4 | 13.6 | 14.1 | 21.6 | 23.9 | 25.1 | 26.9 | 31.7 | 29.9 |

(D) SANGER

| ASSIGNMENT CATEGORIES | SPECIES UNKNOWN | | | GENUS UNKNOWN | | | FAMILY UNKNOWN | | |
|---|---|---|---|---|---|---|---|---|---|
| | DI | SI | ME | DI | SI | ME | DI | SI | ME |
| HIGHER LEVELS | 4.6 | 4.7 | 9.7 | 11.1 | 15.7 | 20.2 | 11.1 | 12.8 | 30.3 |
| INTERMEDIATE LEVELS | 56.3 | 45.3 | 16.0 | 64.4 | 54.2 | 30.6 | 70.1 | 50.8 | 22.2 |
| SPECIFIC LEVELS | 29.2 | 31.6 | 40.0 | 5.7 | 6.1 | 8.4 | 0.0 | 0.0 | 0.0 |
| TOTAL CORRECT ASSIGNMENTS | 90.1 | 81.6 | 65.7 | 81.2 | 76.0 | 59.2 | 81.2 | 63.6 | 52.5 |
| WRONG | 1.3 | 2.7 | 31.4 | 3.7 | 5.9 | 29.2 | 3.7 | 8.2 | 35.3 |
| UNASSIGNED + NO HITS | 8.6 | 15.8 | 2.9 | 15.1 | 18.2 | 11.6 | 15.1 | 28.3 | 12.2 |

'Species Unknown', 'Genus Unknown' and 'Family Unknown' refer to the database variants used. A detailed description of the database variants is given in the Results section of the manuscript. Note that the subtotals may vary by a value of 0.1, since the individual values are rounded off to single decimals.
